# Abdominal Obesity (Waist‐to‐Hip Ratio) is Superior to Body Mass Index as an Indicator of Cognitive Health in Older African Americans

**DOI:** 10.1002/alz.091439

**Published:** 2025-01-09

**Authors:** Sophee Niraula, Bernadette A. Fausto, Zuzanna Osiecka, Mark A. Gluck

**Affiliations:** ^1^ Rutgers University–Newark, Newark, NJ USA

## Abstract

**Background:**

Recent studies suggest that body mass index (BMI) may overestimate obesity and related cognitive health consequences in some populations due to racial differences in body shape and composition. Conversely, waist‐to‐hip‐ratio (WHR) may better capture cognitive health, because this measure accounts for where body fat accumulates. However, few studies have compared the influence of BMI vs. WHR on cognitive function in older African Americans. As such, this study aimed to identify the relative contributions of BMI and WHR to cognitive function in older African Americans.

**Method:**

122 older African American participants (mean age = 72.19; SD = ±6.65) from the *Pathways to Healthy Aging in African Americans* study completed demographic and mood questionnaires, cognitive assessments of attention, memory, executive function, and language, and anthropometric measurements (height, weight, waist and hip circumference). Participants were categorized into four BMI groups per established cut offs (normal weight = 18.5 to 24.9 kg/m^2^; overweight = 25 to 29.9 kg/m^2^; class 1 obese = 30.0 to 34.9 kg/m^2^; class 2 obese or greater = > 35.0 kg/m^2^). We also used the World Health Organization’s proposed cutoffs for WHR to classify participants with and without abdominal obesity (men ≥ 0.90 cm; women ≥ 0.85 cm). ANCOVA was employed to investigate the main effects of BMI and WHR groups while controlling for age, sex, education, depressive symptomatology, and APOE‐e4 carrier status.

**Result:**

The WHR group with abdominal obesity had significantly poorer attention and executive function performance via the Digit Span test, *F*(1, 75) = 7.37, *p* = 0.01, η^2^_p_ = .09). There were no significant differences in cognitive test performance across the BMI groups when controlling for WHR, *F*(3, 75) = 54.10, *p* = .12, η^2^_p_ = .07.

**Conclusion:**

Abdominal obesity (WHR) is superior to BMI as an indicator of cognitive health in older African Americans. The lack of independent association between BMI and cognition highlights the importance of considering more nuanced measures of body weight such as WHR and abdominal obesity in older African Americans

